# Determining ED90 of Flumazenil for Selective Respiratory Distress Improvement Using Remimazolam During Endoscopic Submucosal Dissection of Gastric Neoplasms: A Prospective Study

**DOI:** 10.3390/cancers17020321

**Published:** 2025-01-20

**Authors:** Hyun Il Kim, Da Hyun Jung, Sung Jin Lee, Namo Kim, Seung Hyun Kim, Yu Jun Ji, Hyo-Jin Byon, Sung Kwan Shin

**Affiliations:** 1 Department of Anesthesiology and Pain Medicine, Severance Hospital, Yonsei University College of Medicine, 50-1 Yonsei-ro, Seodaemun-gu, Seoul 03722, Republic of Korea; choco8926@yuhs.ac (H.I.K.); sj122345@yuhs.ac (S.J.L.); namo@yuhs.ac (N.K.); anesshkim@yuhs.ac (S.H.K.); wldbwns@gmail.com (Y.J.J.); 2 Division of Gastroenterology, Department of Internal Medicine, Severance Hospital, Yonsei University College of Medicine, 50-1 Yonsei-ro, Seodaemun-gu, Seoul 03722, Republic of Korea; jungdh@yuhs.ac

**Keywords:** flumazenil, remimazolam, respiratory distress, monitored anesthesia care, sedation, sedation reversal, endoscopic submucosal dissection, anesthesia safety

## Abstract

Background: Patients undergoing endoscopic submucosal dissection under monitored anesthesia care (MAC) with remimazolam may develop respiratory distress during the procedure. In these cases, low doses of flumazenil improved respiratory distress without completely reversing sedation, which is a novel phenomenon. This study aimed to explore the ED90 of flumazenil to selectively improve respiratory distress in patients with MAC treated with remimazolam. Methods: Flumazenil dose determination followed a biased-coin up-and-down design. Starting with a dose of 5 mcg, if respiratory distress improved, the biased-coin method was used to give the same dose in the next patient with a probability of 8/9, and a decreased dose of 5 mcg in the next patient with a probability of 1/9. Any improvement in respiratory distress within 30 s of flumazenil administration was recorded. After the procedure, patients were asked whether they had any memory recall during the procedure. Centered isotonic regression was used to determine the ED90 of flumazenil. Results: Sixty patients were included in the study. The estimated ED90 was 76.72 mcg (95% CI: 68.07–102.62). Memory recall occurred in two of thirteen patients (15%) near the ED90 dose range (75 mcg and 80 mcg). None of the patients developed major postoperative complications (bleeding, perforation, or aspiration) within the 2-day postoperative period. Conclusions: This study determined that the ED90 of flumazenil for effectively alleviating respiratory distress in patients undergoing MAC with remimazolam was 76.7 mcg, without reversing consciousness. These findings provide valuable guidance for the care of patients undergoing sedation.

## Simple Summary

This study explores the optimal dose of flumazenil to address respiratory distress in patients undergoing sedation with remimazolam during endoscopic submucosal dissection (ESD). When monitored anesthesia care (MAC) is performed with remimazolam, a benzodiazepine, respiratory distress caused by excessive sedation can be resolved by administering flumazenil, a benzodiazepine reversal agent. However, this also reverses the sedative state, potentially disrupting the procedure and requiring additional time to re-establish sedation. By determining the effective dose of flumazenil that alleviates respiratory issues without interrupting sedation, this research provides valuable insights for clinicians. The result of this study determined that the ED90 of flumazenil for selectively alleviating respiratory distress in patients undergoing MAC with remimazolam during endoscopic submucosal dissection (ESD), without reversing consciousness, was 76.7 mcg. The findings will help enhance patient safety during procedures requiring sedation, especially those performed under MAC.

## 1. Introduction

Monitored anesthesia care (MAC) is an anesthesia management approach used for surgeries or procedures that neither involve significant surgical stimulation nor require general anesthesia via intubation [1,2,3]. Remimazolam, a novel ultrashort-acting benzodiazepine that has been recently used in clinical practice, has the advantage of causing fewer hemodynamic changes and fewer respiratory disturbances in patients compared to propofol and midazolam and can be reversed quickly with flumazenil, a specific benzodiazepine receptor antagonist [4,5,6]. These characteristics are particularly beneficial for patients who are not intubated and undergo MAC procedures.

Endoscopic submucosal dissection (ESD) is advantageous in treating early gastric neoplasms, providing quick recovery, stomach preservation, and reduced hospitalization, thereby enhancing quality of life and yielding favorable oncologic outcomes [7,8,9]. At our center, remimazolam is widely used for sedation in patients undergoing ESD. A previous study demonstrated that MAC for ESD procedures was effectively achieved by administering intravenous infusion of remimazolam and appropriate fentanyl dosing based on the patient characteristics, representing the standard MAC regimen for ESD procedures at our institution [10].

Considerations for MAC in ESD procedures include the challenge of quickly securing the airway, given that the endoscope is inserted through the mouth. Moreover, the painful nature of dissecting gastric neoplasms requires deeper sedation and analgesia than other diagnostic endoscopic procedures, increasing the risk of respiratory distress due to the high doses of sedatives and opioid doses. Together, this puts the patient at a high risk of hypoxemia due to the difficulty in rapid airway securement.

The general treatment for respiratory distress during MAC is to discontinue sedation and opioids first and assist ventilation with oxygen using maneuvers such as jaw thrusts or airway device insertion to secure the airway. When MAC is performed with remimazolam, the benzodiazepine reversal agent, flumazenil, can be used to awaken the patient from anesthesia in the event of acute respiratory distress. However, fully awakening the patient mid-procedure presents practical difficulties, including interrupting the procedure, inconveniencing the patient and operator, and requiring re-anesthesia to resume the procedure.

In our experience, when respiratory distress occurs due to remimazolam overdose during MAC for ESD, flumazenil administered at small doses below the standard dose for full awakening can alleviate respiratory distress without returning the patient to consciousness. To the best of our knowledge, this effect of flumazenil, specifically in improving respiratory distress at low doses, has not been previously reported. If more than necessary flumazenil is administered, the patient will return to full consciousness, whereas improvement in respiratory distress will not be achieved with too little flumazenil. Therefore, it is crucial to determine the precise dose that selectively alleviates respiratory distress without fully awakening the patient. Should this study provide evidence for the novel effects of flumazenil, it is anticipated to significantly benefit clinical practice for patients undergoing MAC procedures using benzodiazepine-based sedatives such as remimazolam. Thus, this study aimed to determine the ED90 of flumazenil that ameliorates respiratory distress during MAC with remimazolam in patients undergoing ESD.

## 2. Materials and Methods

### 2.1. Patients

This prospective single-center study was conducted at a tertiary hospital. The study protocol was approved by the Institutional Review Board of Severance Hospital, Yonsei University Health System (approval number: 2022-4348-001) and registered at ClinicalTrials.gov (NCT06563063). This study was designed and conducted following CONSORT 2010 guidelines. Patients were informed about the study before ESD and enrolled after obtaining informed consent. The inclusion criteria were as follows: (1) American Society of Anesthesiologists (ASA) class I-III, 19 years of age or older, scheduled for gastric ESD; (2) scheduled to undergo MAC with remimazolam; (3) hemodynamically stable; and (4) development of respiratory distress during the procedure. The exclusion criteria were as follows: (1) the presence of airway-related anatomic abnormalities and (2) administration of any sedative other than remimazolam during the procedure (e.g., propofol or dexmedetomidine). Patients fully awakened from anesthesia for any other reason during the procedure were planned to be excluded from the study.

### 2.2. Procedure

Blood pressure and heart rate measured in the pre-procedure room were defined as baseline vital signs. After the patient was admitted to the procedure room, routine monitoring (noninvasive blood pressure cuff, electrocardiogram, and pulse oximetry) was performed. Remimazolam (Byfavo inj, remimazolam besylate, 20 mg/vial, Hana Pharm Co., Ltd., Seoul, Republic of Korea) 5 mg dosage was administered intravenously over 1 min to initiate sedation, followed by a continuous intravenous infusion of remimazolam at a rate of 0.1 to 0.4 mg∙kg^−1^∙h^−1^ during the procedure, aiming for a Modified Observer’s Assessment of Alertness/Sedation Scale (MOAA/S) score of 2–3. Fentanyl (Fentanyl inj, fentanyl citrate, 100 mcg/2 mL/ampule, Hana Pharm Co., Ltd., Seoul, Republic of Korea) 50 mcg dosage was initially administered with remimazolam for pain control.

There were a total of five endoscopists involved in this study. When the MAC induction agent was injected, an endoscope was inserted, the lesion location was confirmed, and circumferential marking was performed. Submucosal injections of a solution consisting of epinephrine (0.01 mg/mL), 0.8% indigo carmine, and normal saline were administered and a mucosal incision was made, followed by submucosal layer dissection. A dual knife (KD-650Q; Olympus, Tokyo, Japan) or an insulated-tip knife (KD-610L; Olympus Optical, Tokyo, Japan) was used for lesion incision and dissection. After ESD, the bleeding site was ablated using hemostatic forceps (Coagrasper; Olympus Co., Tokyo, Japan), and the procedure was completed.

During the procedure, if the patient’s blood pressure increased (>20% of baseline systolic blood pressure), pulse rate increased (>20% of baseline heart rate), or the patient moved, the patient was considered to have responded to the pain stimulus, and another bolus of 25 mg fentanyl was administered. Respiratory distress occurring during the procedure was defined as follows: (1) sustained hypoxia (SpO_2_ < 94%) lasting more than 15 s, or (2) excessive irregular breathing with heavy chest and abdominal movements, including hiccups, which made it difficult for the endoscopist to perform the procedure, as determined by the endoscopist’s judgment. In the event of respiratory distress, flumazenil (Lumasate inj, flumazenil, 0.5 mg/5 mL/ampule, Hana Pharm Co., Ltd., Seoul, Republic of Korea) was administered, and the patient was monitored. The dose level set in this study (5 mcg) was highly particular compared to the original concentration of flumazenil (0.1 mg/mL, i.e., 100 mcg/mL). Therefore, to ensure accurate dosing of flumazenil, flumazenil was pre-diluted to one-tenth of its original concentration before the procedure. This dilution yielded a solution where the desired dose level corresponded to 0.5 mL of the diluted solution. Improvement in respiratory distress was defined as the recovery of hypoxia (SpO_2_ ≥ 95%) and improvement in respiratory pattern—loss of excessively irregular breathing and subdued chest and abdominal movement—within 30 s after flumazenil administration to the extent that the procedure could be resumed.

### 2.3. Dose Level Determination: Biased-Coin Up-and-Down Design

The dosing of flumazenil followed a biased-coin up-and-down design, which is widely used in dose-finding studies [11,12,13,14,15]. The biased up-and-down method, an application of the up-and-down method first proposed by Dixon et al., can achieve robust results with significantly fewer samples compared to older dose-finding experiment designs [16,17]. Starting with an initial dose of 5 mcg, if there was no improvement, the next patient was given a 5 mcg increase. If respiratory distress improved, the biased-coin method was used to administer the same dose to the next patient with a probability of 8/9 and a decreased dose of 5 mcg to the next patient with a probability of 1/9. To determine the probability, a random number generator randomly generated a number from 1 to 9. If the randomly generated number was 1 to 8, the dose was maintained, and if the number was 9, the dose was reduced. As no previous studies were available to guide the setting of the dose range, we set the dose range based on doses that have been used empirically to improve respiratory distress without awakening consciousness at our institution. The minimum level of dose range was 5 mcg and the maximum level was 150 mcg.

If there was no respiratory improvement with flumazenil treatment, conventional airway management was initiated, including a 30% reduction in the remimazolam rate, jaw thrust, and insertion of a nasopharyngeal airway device if respiratory distress persisted. Once the respiratory distress improved, the procedure was resumed. At the end of the procedure, patients were moved to the recovery unit and asked whether they had any memory recall during the procedure. The primary endpoint of the study was the dose of flumazenil required to improve respiratory distress, and the secondary endpoint was the number of recall events during sedation. Other patient demographics, clinicopathological characteristics of the neoplasms, and major postoperative complications (bleeding, perforation, and aspiration) within the 2-day postoperative period were also collected.

### 2.4. Statistical Analysis

**Figure 1:**
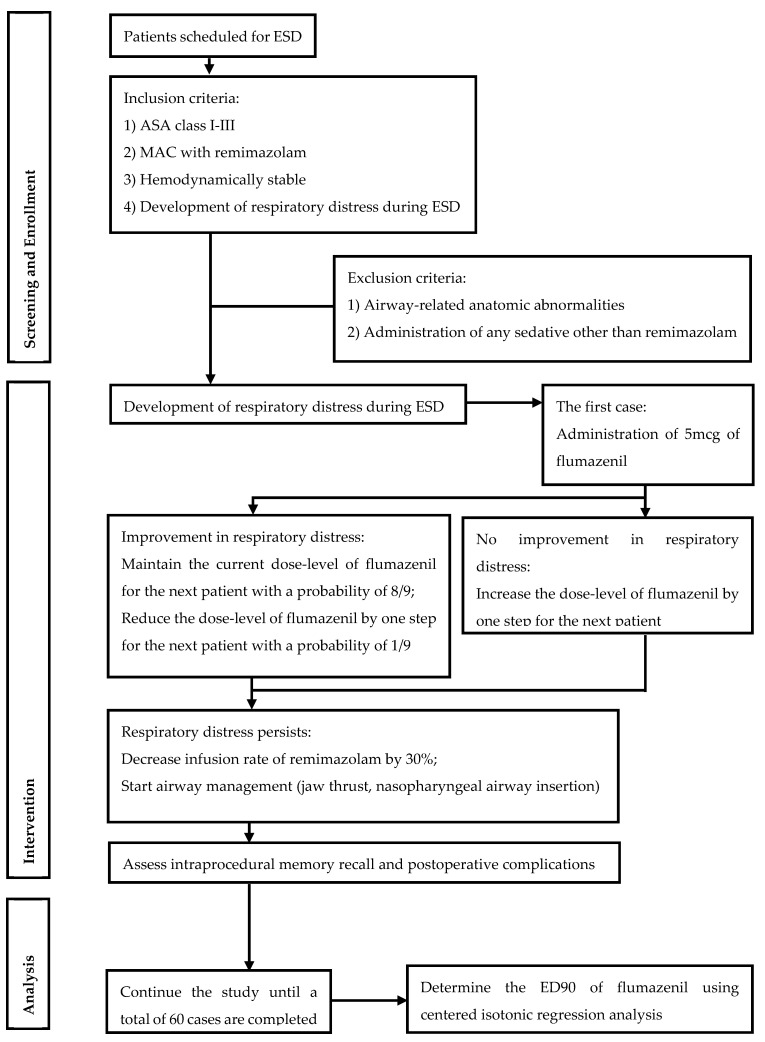
Schematic diagram of the study. ESD, endoscopic submucosal dissection; ASA, American Society of Anesthesiologists; MAC, monitored anesthesia care; ED90, 90% effective dose.

According to a recently published paper, the recommended number of samples for obtaining the ED90 by a biased-coin up-and-down design is between 50 and 60 [11]. Based on this guidance, we set the required sample size at 60 for the statistical analysis. Using data from all 60 cases, the ED90 was determined through centered isotonic regression, a method employed in dose-finding studies, where the proportion of patients who responded at each dose level is used to obtain a cumulative distribution function of the proportion of patients responding to the dose [17]. This method was used to determine the point estimate and the 95% confidence interval of the ED90 of flumazenil for improving respiratory distress. The probability of the secondary endpoint (intraprocedural recall) at ED90 was calculated by assessing the proportion of patients with a recall at dose levels immediately above and below ED90. The overall flow of this study, as described above, is illustrated in the schematic diagram provided in Figure 1.

## 3. Results

### 3.1. Patient Demographics

Informed consent was obtained from 132 patients before ESD, of whom 60 (45%) developed respiratory distress during the procedure. The demographic data of the 60 patients are shown in Table 1. The mean age of the patients was 66.7 years and 63.3% were male. The proportions of ASA classes I, II, and III were 11.7%, 80.0%, and 8.3%, respectively.

The pathological characteristics of the neoplasms were also recorded. The locations of the neoplasms were divided into three groups with the number of cases in each group as follows: upper (cardia, fundus)—5 (8.3%); mid (body)—14 (23.3%); and lower (antrum, pylorus)—41 (68.3%). The median length of the neoplasm, measured using endoscopy, was 15 mm. Histology of the neoplasms was confirmed by pathology and categorized into four groups, with the number of cases in each group as follows: I, dysplasia (epithelial dysplasia, low or high grade)—34 (56.7%); II, differentiated cancer (tubular adenocarcinoma, well or moderately differentiated)—16 (26.7%); III, undifferentiated cancer (tubular adenocarcinoma, poorly differentiated; gastric carcinoma, poorly cohesive or signet ring cell type)—6 (10.0%); and IV, other (neuroendocrine tumor, etc.)—4 (6.7%).

The median anesthesia time was 30 min. Additionally, the mean total dosage was 14.8 mg for remimazolam and 118.8 mcg for fentanyl. Detailed demographic data and outcomes of all patients are attached as a Supplementary File Table S1.

### 3.2. ED90 of Flumazenil for Remimazolam to Alleviate Respiratory Distress During ESD

**Figure 2:**
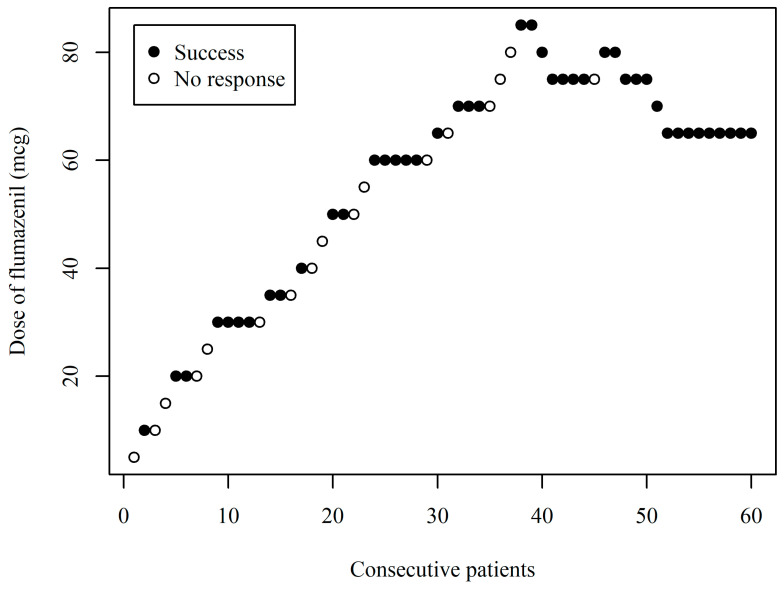
Responses (improvement of respiratory distress) of 60 consecutive patients who received flumazenil for improving respiratory distress while maintaining sedation under MAC with remimazolam during the ESD procedure.

**Figure 3:**
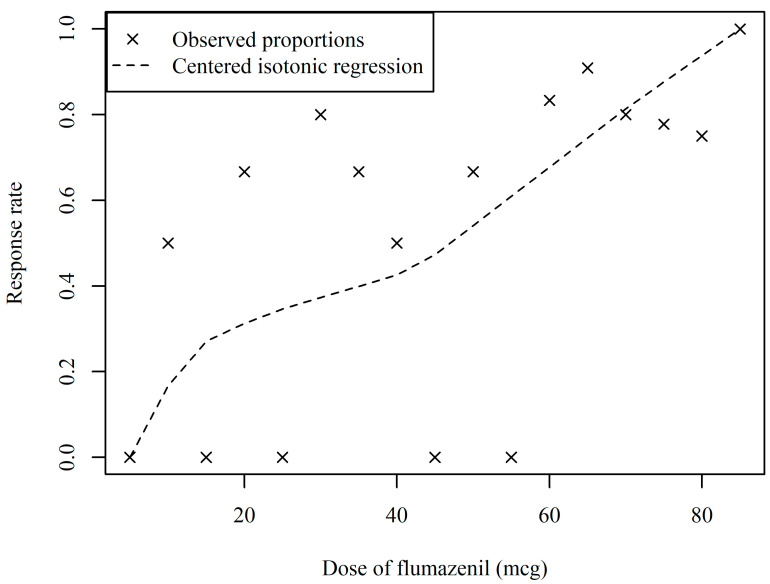
Actual observed proportions of success in improving respiratory distress at each dose level and the cumulative distribution curve calculated using centered isotonic regression based on observed proportions.

The sequence of the patient response results obtained using the biased-coin up-and-down method is shown in Figure 2. The dose of flumazenil was varied within a range of 5 to 85 mcg. The estimated ED90 from the data was 76.72 mcg (95% CI: 68.07–102.62). The estimated centered isotonic regression model is shown in Figure 3.

### 3.3. Secondary Endpoints

Among the total patients, four patients developed memory recall (7%): one at 70 mcg, two at 80 mcg, and one at 85 mcg. Recall occurred in two of the thirteen patients (15%) at dose levels adjacent to ED90. None of the patients developed major postoperative complications (bleeding, perforation, or aspiration) within the 2-day postoperative period.

## 4. Discussion

In this study, we explored the ability of the ED90 of flumazenil to selectively improve respiratory distress without fully restoring consciousness during the procedure in patients undergoing ESD under MAC with remimazolam. The estimated ED90 of flumazenil for this effect was 76.72 mcg, and memory recall at ED90 occurred in 2 of 13 patients (15%). The findings of this study substantiate the novel effect of flumazenil to improve respiratory distress only at low doses, offering significant clinical benefits in sedation procedures in which respiratory distress may occur unpredictably.

The primary aim of this study was to investigate the ED90 of flumazenil, which improves respiratory distress in patients undergoing ESD under MAC with remimazolam. This means that administering flumazenil at the ED90 to patients experiencing respiratory distress in such conditions will result in significant alleviation of respiratory distress in 90% of such patients. During endoscopic procedures, such as ESD with MAC, respiratory distress can occur at any time if a sedative agent is administered without an invasive airway. This dangerous situation can lead to hypoxia, resulting in serious complications if not managed appropriately. Moreover, to prevent complications such as bleeding or perforation during ESD, it is crucial for endoscopists to maintain optimal visibility during dissection. Also, deep sedation with remimazolam can lead to respiratory distress characterized by excessive irregular breathing with heavy chest and abdominal movements or hiccups [18]. These respiratory fluctuations may obstruct the endoscopist’s visual field and increase the risk of ESD-related complications. Therefore, reducing respiratory distress is essential to achieve a safe and efficient ESD procedure. Additionally, if the maneuverability of the scope is unstable due to this paradoxical movement, the procedure time may significantly increase [19]. Therefore, if respiratory distress occurs during sedation, immediate action is required, and flumazenil, at its ED90, can be administered to improve the patient’s breathing and prevent hypoxia.

Benzodiazepines exert sedative effects by acting on gamma-aminobutyric acid (GABA)_A_ receptors to enhance GABA, a crucial inhibitory neurotransmitter in the central nervous system [20]. Flumazenil serves as an antidote to the benzodiazepines’ sedative effects by functioning as a competitive antagonist [21]. Currently, it remains unclear why a small dose of flumazenil selectively improves respiratory distress in patients sedated with remimazolam, although we can propose a hypothesis. Remimazolam typically provides reliable sedation at standard doses, with respiratory depression occurring in only a few patients [22,23]. This suggests that, while consciousness can be effectively suppressed, respiratory function is less prone to suppression from remimazolam administration. Therefore, when sedation reaches a depth sufficient to induce both unconsciousness and respiratory depression, a low dose of flumazenil may potentially reduce sedation to a level where consciousness is still suppressed while restoring the function of the respiratory center. This hypothesis is supported by the findings of this study, though further well-designed research is necessary to ascertain the precise mechanism underlying this effect.

In this study, the initial dose of flumazenil and the incremental dose differences between levels were set at 5 mcg. These doses were determined arbitrarily due to the absence of prior studies. Based on our clinical experience before designing the study, we observed that a dose of 10 to 20 mcg of flumazenil often improved respiratory distress in many patients. If no improvement occurred with the first dose, a second dose of the same amount was frequently administered. Given these experiences, we anticipated that the ED90 of flumazenil would fall within the range of 20 to 40 mcg, and thus decided to increment dose levels by 5 mcg to obtain precise data. Additionally, we did not calculate flumazenil doses based on body weight for the following reasons: Dosing in 5 mcg increments, given its common availability as 100 mcg/cc, would necessitate complex drug dosing calculations. Dividing doses by body weight would further require calculations in 0.1 mcg increments, making dosing impractical and less feasible in a clinical setting.

All patients enrolled underwent ESD with MAC using remimazolam and fentanyl, the standard sedation approach for ESD. As previously mentioned [4,5,6], remimazolam is associated with fewer hemodynamic changes and less respiratory depression than other sedatives, making it a suitable choice for MAC. We used fentanyl rather than ultrashort-acting remifentanil for analgesia due to the unpredictable nature of ESD procedures, which makes estimating their duration challenging. In shorter cases, an initial fentanyl bolus provided sufficient analgesia. Depending on the course of the procedure, painful stimuli of high intensity may occur, resulting in sudden spontaneous patient movement [24]. In such instances, an additional fentanyl bolus effectively managed the pain. Administering a bolus of remifentanil in response to sudden severe pain is likely to induce apnea. Therefore, we preferred fentanyl to remifentanil as the standard opioid. Caution is advised when extrapolating the results of this study to those of sedation using other agents.

At higher doses, flumazenil may completely reverse the sedative effects of remimazolam, potentially leading to postprocedural memory recall. In this study, postprocedural memory recall occurred in 4 of the 60 patients (7%). However, given the risk of respiratory distress during MAC, prioritizing the maintenance of patient respiration is more critical than preventing memory recall in a small number of cases. Additionally, all patients who experienced recall had successful procedures, and no major adverse events were reported, suggesting that recall did not impact their overall prognosis.

This study had several limitations. First, this was a single-center study conducted exclusively on single-race patients (Asian) undergoing ESD with MAC, using specific agents such as remimazolam and fentanyl. Consequently, caution should be exercised when generalizing these results to other sedative practices. Second, providing highly objective criteria for determining respiratory distress during sedation and assessing whether patients experienced memory recall remains challenging. We used pulse oximetry to detect hypoxia and monitored irregular breathing as well as heavy chest and abdominal movements that could interfere with the procedure. However, evaluating the extent of irregular breathing and movement involves a degree of subjective judgment by physicians. Furthermore, since memory recall during the procedure ultimately relies on the patient’s self-report, establishing a strictly objective evaluation is challenging. Third, the use of fentanyl and remimazolam during sedation may have contributed to respiratory distress because opioids can also affect respiration. Nonetheless, fentanyl was administered as a small bolus dose to address inadequate analgesia, thereby minimizing its potential impact on respiration. Finally, the confidence interval for the ED90 estimate is relatively wide (ED90: 76.72 mcg; 95% CI: 68.07–102.62). Despite this, this study provides valuable insights into the previously unrecognized effects of flumazenil, and we are planning additional studies to determine a more accurate ED90 and to explore the clinical utility of the newly identified effects of flumazenil uncovered in this research.

## 5. Conclusions

This study determined that the ED90 of flumazenil for selectively alleviating respiratory distress in patients undergoing monitored anesthesia care (MAC) with remimazolam during endoscopic submucosal dissection (ESD), without reversing consciousness, was 76.7 mcg. Additional studies involving larger and more racially diverse populations will be needed to more accurately determine the ED90 of flumazenil. The findings of this paper highlight a novel clinical application of flumazenil that, to the best of our knowledge, has not been previously reported. Specifically, the ability of flumazenil to selectively alleviate respiratory distress could significantly enhance the clinical management of patients undergoing MAC with remimazolam.

## Figures and Tables

**Table 1:** Patient demographic data.

Variables	Value
Age, mean (SD), yr	66.7 (8.9)
Sex	Female: 22 Male: 38
Weight, mean (SD), kg	67.3 (10.5)
Height, mean (SD), cm	164.0 (8.8)
BMI, mean [range] (SD), kg∙m^−2^,	25.0 [18.7–37.7] (3.0)
ASA class	I: 7 II: 48 III: 5
Location	Upper: 5 Mid: 14 Lower: 41
Length of neoplasm measured by endoscopy, median (interquartile range), mm	15 (10–20)
Histology	I: 34 II: 16 III: 6 IV: 4
Anesthesia time, median (interquartile range), min	30 (20–40)
Total remimazolam dose, mean (SD), mg	14.8 (5.6)
Total fentanyl dose, mean (SD), mcg	118.8 (67.0)

BMI, body mass index; ASA, American Society of Anesthesiologists. The location groups were upper (cardia and fundus), middle (body), and lower (antrum and pylorus). The histology groups were as follows: I, dysplasia (epithelial dysplasia, low or high grade); II, differentiated cancer (tubular adenocarcinoma, well or moderately differentiated); III, undifferentiated cancer (tubular adenocarcinoma, poorly differentiated; gastric carcinoma, poorly cohesive or signet ring cell type); and IV, other (neuroendocrine tumor).

## Data Availability

The data are not publicly available due to privacy of research participants.

## Supplementary Materials

The following supporting information can be downloaded at: https://www.mdpi.com/article/10.3390/cancers17020321/s1, Table S1. Detailed information on all subjects participating in the study.

## References

[1] Smith I. (1996). Monitored anesthesia care: How much sedation, how much analgesia?. J. Clin. Anesth..

[2] Rego M.M.S., Watcha M.F., White P.F. (1997). The changing role of monitored anesthesia care in the ambulatory setting. Anesth. Analg..

[3] Nonaka S., Kawaguchi Y., Oda I., Nakamura J., Sato C., Kinjo Y., Abe S., Suzuki H., Yoshinaga S., Sato T. (2015). Safety and effectiveness of propofol-based monitored anesthesia care without intubation during endoscopic submucosal dissection for early gastric and esophageal cancers. Dig. Endosc..

[4] Wesolowski A.M., Zaccagnino M.P., Malapero R.J., Kaye A.D., Urman R.D. (2016). Remimazolam: Pharmacologic considerations and clinical role in anesthesiology. Pharmacother. J. Hum. Pharmacol. Drug Ther..

[5] Chen S., Wang J., Xu X., Huang Y., Xue S., Wu A., Jin X., Wang Q., Lyu J., Wang S. (2020). The efficacy and safety of remimazolam tosylate versus propofol in patients undergoing colonoscopy: A multicentered, randomized, positive-controlled, phase III clinical trial. Am. J. Transl. Res..

[6] Sneyd J.R., Rigby-Jones A.E. (2020). Remimazolam for anaesthesia or sedation. Curr. Opin. Anesthesiol..

[7] Lian J., Chen S., Zhang Y., Qiu F. (2012). A meta-analysis of endoscopic submucosal dissection and EMR for early gastric cancer. Gastrointest. Endosc..

[8] Tanabe S., Ishido K., Higuchi K., Sasaki T., Katada C., Azuma M., Naruke A., Kim M., Koizumi W. (2014). Long-term outcomes of endoscopic submucosal dissection for early gastric cancer: A retrospective comparison with conventional endoscopic resection in a single center. Gastric Cancer.

[9] Bourke M.J., Neuhaus H., Bergman J.J. (2018). Endoscopic submucosal dissection: Indications and application in western endoscopy practice. Gastroenterology.

[10] Kim H.I., Jung D.H., Lee S.J., Lee Y.C., Lee S.K., Kim G.H., Nam H.J., Lee S., Byon H.-J., Shin S.K. (2024). Associations between Clinicopathological Characteristics and Intraoperative Opioid Requirements during Endoscopic Submucosal Dissection with Monitored Anesthesia Care: A Retrospective Study. J. Clin. Med..

[11] Oron A.P., Souter M.J., Flournoy N. (2022). Understanding research methods: Up-and-down designs for dose-finding. Anesthesiology.

[12] Choi S.H., Min K.T., Lee J.-R., Choi K.W., Han K.-H., Kim E.H., Oh H.J., Lee J.H. (2015). Determination of EC95 of remifentanil for smooth emergence from propofol anesthesia in patients undergoing transsphenoidal surgery. J. Neurosurg. Anesthesiol..

[13] Kanczuk M.E., Barrett N.M., Arzola C., Downey K., Xiang Y.Y., Carvalho J.C. (2017). Programmed intermittent epidural bolus for labor analgesia during first stage of labor: A biased-coin up-and-down sequential allocation trial to determine the optimum interval time between boluses of a fixed volume of 10 mL of bupivacaine 0.0625% with fentanyl 2 μg/mL. Anesth. Analg..

[14] Au K., Shippam W., Taylor J., Albert A., Chau A. (2020). Determining the effective pre-oxygenation interval in obstetric patients using high-flow nasal oxygen and standard flow rate facemask: A biased-coin up–down sequential allocation trial. Anaesthesia.

[15] Wesselink E.J., Koopman S.J., van der Vegt R., van de Ven P.M., van der Aa J.P., Stapper C., Wesdorp F., de Kok L., Zhang Y., Franssen E.J. (2022). ED90 of spinal 2-chloroprocaine 1% in ambulatory knee arthroscopy up to 45 min: A randomized biased-coin-up-and-down sequential allocation trial. Reg. Anesth. Pain Med..

[16] Dixon W.J., Mood A.M. (1948). A method for obtaining and analyzing sensitivity data. J. Am. Stat. Assoc..

[17] Oron A.P., Flournoy N. (2017). Centered isotonic regression: Point and interval estimation for dose–response studies. Stat. Biopharm. Res..

[18] Liu C.C., Lu C.Y., Changchien C.F., Liu P.H., Perng D.S. (2012). Sedation-associated hiccups in adults undergoing gastrointestinal endoscopy and colonoscopy. World J. Gastroenterol. WJG.

[19] Hamada K., Horikawa Y., Koyanagi R., Shiwa Y., Techigawara K., Nishida S., Nakayama Y., Honda M. (2019). Usefulness of a multibending endoscope in gastric endoscopic submucosal dissection. VideoGIE Off. Video J. Am. Soc. Gastrointest. Endosc..

[20] Greenblatt D.J. (1992). Pharmacology of benzodiazepine hypnotics. J. Clin. Psychiatry.

[21] Whitwam J., Amrein R. (1995). Pharmacology of flumazenil. Acta Anaesthesiol. Scand..

[22] Oka S., Satomi H., Sekino R., Taguchi K., Kajiwara M., Oi Y., Kobayashi R. (2021). Sedation outcomes for remimazolam, a new benzodiazepine. J. Oral Sci..

[23] Jhuang B.-J., Yeh B.-H., Huang Y.-T., Lai P.-C. (2021). Efficacy and safety of remimazolam for procedural sedation: A meta-analysis of randomized controlled trials with trial sequential analysis. Front. Med..

[24] Park C.H., Shin S., Lee S.K., Lee H., Lee Y.C., Park J.C., Yoo Y.C. (2015). Assessing the stability and safety of procedure during endoscopic submucosal dissection according to sedation methods: A randomized trial. PLoS ONE.

