# Supplementary material for: Determining ED90 of Flumazenil for Selective Respiratory Distress Improvement Using Remimazolam During Endoscopic Submucosal Dissection of Gastric Neoplasms: A Prospective Study
Source: Cancers (Basel). 2025 Jan 20;17(2):321. doi: 10.3390/cancers17020321 (PMC11763922; doi:10.3390/cancers17020321)
Supplement: Supplementary file 1 [file cancers-17-00321-s001.zip › cancers-3395337-supplementary.pdf]

Supplementary Table S1. Detailed information on all subjects participating in the study

Case	Age	Sex	Weight (kg)	Height (cm)	BMI (kg/m^2)	ASA	Location	Length (mm)	Histology	Anesthesia time (min)	Flumazenil dose (mcg)	Remimazolam dose (mg)	Fentanyl dose (mcg)	Success	Recall
1	77	M	74.6	168.9	26.2	2	Upper	15	IV	20	5	11	100	0	0
2	64	M	77.3	173.4	25.7	2	Mid	10	I	15	10	10	50	1	0
3	80	M	69.9	171.7	23.7	2	Lower	30	I	30	10	13	50	0	0
4	70	M	61.4	163.8	22.9	2	Lower	15	I	20	15	11	50	0	0
5	64	M	76.9	171.5	26.1	2	Mid	10	I	95	20	24	200	1	0
6	62	M	77.4	171.4	26.3	2	Lower	20	I	25	20	12	100	1	0
7	65	F	56.4	147.1	26.1	2	Lower	8	II	25	20	11	50	0	0
8	56	F	57.8	156.4	23.6	2	Lower	20	I	35	25	13	50	0	0
9	62	M	83.6	182.3	25.2	2	Lower	10	I	30	30	16	100	1	0
10	74	F	55.8	155.8	23	2	Lower	10	II	30	30	13	150	1	0
11	74	M	77.1	167.8	27.4	2	Lower	15	II	15	30	10	100	1	0
12	78	F	63	161.2	24.2	2	Lower	10	I	20	30	11	100	1	0
13	62	M	60.7	168.2	21.5	2	Lower	40	III	30	30	12	50	0	0
14	63	F	59.5	154.6	24.9	2	Mid	25	IV	20	35	11	50	1	0
15	78	M	69.3	165.9	25.2	2	Lower	20	II	35	35	15	150	1	0
16	68	M	67.2	170.4	23.1	2	Lower	10	II	35	35	12	75	0	0
17	76	F	65.1	153.7	27.6	2	Lower	40	II	40	40	15	150	1	0
18	74	M	63.3	161.8	24.2	2	Lower	20	I	30	40	11	150	0	0
19	68	M	77.7	168.7	27.3	2	Mid	20	I	40	45	13	100	0	0
20	69	F	61.2	154.2	25.7	3	Lower	15	I	30	50	13	100	1	0
21	77	M	76.5	163.3	28.7	3	Lower	15	I	30	50	13	100	1	0
22	58	F	50.1	147.3	23.1	2	Mid	10	III	50	50	17	175	0	0
23	72	M	62.5	168.1	22.1	3	Lower	25	II	60	55	18	75	0	0
24	60	M	86.5	179.5	26.8	2	Upper	10	II	55	60	20	250	1	0
25	66	M	69.6	176.5	22.3	2	Lower	15	I	30	60	13	100	1	0
26	61	M	79.3	171.3	27	2	Upper	20	I	65	60	20	100	1	0
27	64	F	56.9	146.6	26.5	2	Mid	12	III	35	60	14	100	1	0
28	55	M	100	178	31.6	2	Mid	18	I	30	60	12	100	1	0
29	66	M	64.7	172.1	21.8	2	Lower	18	I	25	60	10	100	0	0
30	55	M	59.6	165.9	21.7	2	Mid	19	I	35	65	15	150	1	0
31	75	M	76.3	167.1	27.3	2	Lower	10	I	35	65	16	150	0	0
32	54	M	71.5	160	27.9	1	Lower	18	II	100	70	38	350	1	0
33	75	F	54.9	157.3	22.2	2	Mid	12	I	55	70	20	100	1	0

34	66	F	67.1	156.2	27.5	1	Mid	7	I	15	70	10	100	1	0
35	69	M	68	170.6	23.4	3	Lower	7	I	40	70	16	100	0	0
36	75	M	52.5	167.4	18.7	2	Mid	5	I	20	75	10	50	0	0
37	68	M	56.7	158.3	22.6	2	Lower	12	II	20	80	11	150	0	0
38	66	M	71.2	163.4	26.7	1	Lower	15	I	20	85	11	50	1	0
39	40	F	67.8	160.7	26.3	1	Lower	25	II	125	85	40	400	1	1
40	54	F	63.2	155.6	26.1	1	Mid	3	IV	20	80	11	100	1	1
41	60	M	63	168	22.3	2	Lower	18	II	15	75	11	50	1	0
42	76	F	72.4	164.3	26.8	2	Lower	15	I	30	75	15	100	1	0
43	62	M	60.7	169.7	21.1	2	Lower	10	II	15	75	10	100	1	0
44	76	M	74.2	173	24.8	2	Lower	15	I	30	75	14	75	1	0
45	74	M	76.9	169.8	26.7	2	Upper	20	IV	30	75	12	75	0	0
46	52	M	75.6	167.5	26.9	1	Lower	15	I	50	80	18	150	1	0
47	44	M	81	183.8	24	2	Mid	15	I	45	80	16	200	1	1
48	74	M	65.7	154	27.7	2	Lower	15	I	30	75	14	100	1	0
49	75	F	63	153.3	26.8	2	Lower	15	I	35	75	14	100	1	0
50	67	M	57	157	23.1	2	Lower	25	II	35	75	14	150	1	0
51	58	F	51.4	163.8	19.2	2	Upper	10	III	55	70	20	200	1	1
52	62	F	88.3	153	37.7	2	Lower	15	I	20	65	12	100	1	0
53	78	F	61.6	156.8	25.1	2	Mid	15	I	20	65	11	100	1	0
54	72	F	58.5	155.7	24.1	2	Lower	60	I	65	65	22	200	1	0
55	69	M	66.4	166.2	24	2	Lower	15	I	35	65	15	100	1	0
56	60	F	52.5	158.7	20.8	2	Lower	20	III	40	65	17	200	1	0
57	72	F	62	155	25.8	2	Lower	15	I	20	65	11	100	1	0
58	57	F	45.5	154.9	19	1	Lower	15	II	60	65	18	100	1	0
59	82	M	68.4	164.2	25.4	2	Lower	15	II	45	65	16	150	1	0
60	72	M	81.5	178.8	25.5	3	Lower	10	III	40	65	13	50	1	0

BMI, body mass index; ASA, American Society of Anesthesiologists; Length, length of neoplasm measured by endoscopy. The location groups were upper (cardia and fundus), middle (body), and lower (antrum and pylo-rus). The histology groups were as follows: I, dysplasia (epithelial dysplasia, low or high grade); II, differentiated cancer (tubular adenocarcinoma, well or moderately differentiated); III, undifferentiated cancer (tubular adenocarcinoma, poorly differentiated; gastric carcinoma, poorly cohesive or signet ring cell type); and IV, other (neu-roendocrine tumor).
